# Mr. Pulley, on a Case of Monstrosity

**Published:** 1800-02

**Authors:** John Pulley

**Affiliations:** St. Thomas's Hospital


### *To the Editors of the Medical and Phyfical Journal.*

Gentlemen,

HAVING tranfmitted for the third number of the firft volume of your Journal, the appearances in a cafe of monftrofity, and having a fhort time fince met with a fecond inftance of monftrous production, I am induced to think, that a relation of it may not be uninterefting. The fubject was a full grown female child, and was born dead. The imperfection of the cranial bones, was fimilar to the cafe before related, excepting that the os occipitis was nearly perfect, and its edge was not projected unnaturally outward. The integuments from the occipital bone, did not dip down under its edge, but paffed on the brainy excrefence, and were there foon infenfibly loft. The brain had a fungous appearance, and feemed not to be deficient in quantity; it was not made what is called hemifpherical, by a longitudinal divifion, but appeared a confufed mafs: neither dura nor pia mater apparently exifted. The moft curious part of this monftrofity was the exiftence of a denfe femi-tranfparent membrane, which was attached to the left fide of the fummit of the excrefence, to the extent of about an inch. This membrane paffed down to the left fide of the face, and there formed feveral attachments; it adhered to the upper lip on its left fide, producing a divifion in the lip, which exactly correfponded

---

correſponded in appearance with what is termed the hare-lip; it alſo adhered to the left ala of the noſe, and rendered the noſtril on that ſide, depreſſed and imperforate; it was alſo attached to the integuments on the cheek, juſt under the os malæ; which attachment, with the former, much diſtorted the left ſide of the face; its laſt adheſion, was to the helix of the left ear, juſt above the lobule, which rendered the meatus anditorius externus imperforate, and produced a diviſion of the ear, leaving the lobe ſeparated from the pinna. Perhaps I have expreſſed too much, in aſſerting, that no dura mater apparently exiſted, as Nature *might* originally have intended this membrane to have formed it. The admiſſion into the meatus anditorius externus, through the concha of the right ear, was ſo covered by integument, that the head of a probe could only be introduced; the entrance to the right noſtril was in the ſame way impeded; and the eye-lids of both orbits were completely united, except in having a ſimilar ſmall perforation; and when a probe was paſſed through each perforation into the orbital concavity, it could be paſſed freely about it, and ſeemingly to its bottom; from which I ſhould conclude, that no eyes exiſted.

I am, Gentlemen,

Your's, reſpectfully,

JOHN PULLEY.

*St. Thomas's Hoſpital, Jan.* 10, 1800.

